# Removal of Phosphate from Aqueous Solution Using Alginate/Iron (III) Chloride Capsules: a Laboratory Study

**DOI:** 10.1007/s11270-016-3128-0

**Published:** 2016-10-31

**Authors:** Hanna Siwek, Artur Bartkowiak, Małgorzata Włodarczyk, Katarzyna Sobecka

**Affiliations:** 1 Department of General and Ecological Chemistry, West Pomeranian University of Technology, Szczecin, ul. J. Słowackiego 17, 71-434 Szczecin, Poland; 2 Center of Bioimmobilisation and Innovative Packaging Materials, Faculty of Food Sciences and Fisheries, West Pomeranian University of Technology, Szczecin, ul. K. Janickiego 35, 71-270 Szczecin, Poland

**Keywords:** Alginate, Iron (III) chloride, Phosphorus removal, Surface water, Adsorption

## Abstract

Binding phosphate at participation of alginate/FeCl_3_ capsules was studied with laboratory experiments. The hydrogel microcapsules were obtained with the dropping-in method, by gelation of sodium alginate water solution by iron (III) chloride solution. Phosphate adsorption characteristics were studied in a static batch system with respect to changes in contact time, initial phosphates concentration, pH of solution, and temperature. After 24 h of the tests, average 87.5% of phosphate ions were removed from the natural water solutions; after 48 h, an equilibrium was reached. The adsorption data were well fit by the Freundlich isotherm model. Parameter *k* of the isotherms amounted from 43.4 to 104.7, whereas parameter *n* amounted from 0.362 to 0.476. The course of processes of phosphate adsorption and iron desorption to aquatic phase, as well as changes in pH, suggests that phosphate adsorption is a major mechanism of phosphate removal, whereas simultaneously, but at a much lower degree, a process of precipitation of phosphate by iron (III) ions released from the capsules to the solution takes its place. Parameters calculated in the Freundlich isotherm equation show that by using several times smaller amounts of iron, it is possible to remove similar or bigger amounts of phosphorus than with other adsorbents containing iron. The alginate/FeCl_3_ adsorbent removes phosphate in a wide pH spectrum—from 4 to 10. Results suggest that the proposed adsorbent has potential in remediation of contaminated waters by phosphate.

## Introduction

The research works on reasons and rate of eutrophication of surface waters and contents and transformations of biogenic elements in the natural aquatic environment, which had been conducted for dozens of years, showed that, in the majority of cases, availability of phosphorus is a crucial factor controlling development of planktonic microorganisms (Forsberg and Ryding 1980; White 1989; Correll 1996; Abell et al. 2010). Shortage of phosphorus in the environment leads to a substantial reduction of productivity of water reservoirs, while a surplus generally impedes the process of their eutrophication. Influence of phosphorus on trophic conditions in waters depends not only on its quantity but also on phosphoric compounds management in the entire aquatic ecosystem. Therefore, renaturisation and reclamation of eutrophiced water reservoirs is based, first of all, on reduction of allochtonous and autochtonous deposits of phosphorus. Both in the process of wastewater treatment and inactivation of bioavailable forms of phosphorus in water reservoirs, generally, chemical methods are in use. If phosphorus compounds occur in colloidal arrangements, they are removed in volumetric coagulation process with clear phases of destabilization and flocculation. If phosphorus occurs as a true solution, a process of chemical precipitation takes place. For this purpose, the substances containing the multivalent hydroxides and salts of aluminum (Tandyrak et al. 2001; Hullebusch et al. 2002), calcium (Dittrichm and Koschel 2002), and iron (Perkins and Underwood 2001; Deppe and Benndorf 2002) are primarily used. Apart from many advantages, such as high efficiency and a significant improvement on all stages of wastewater treatment, chemical precipitation has some disadvantages too. For instance, it changes pH of conditioned water and does not provide a possibility to regenerate most of the coagulant effectively, due to which they are recommended rather for single applications (Genz et al. 2004). Because the coagulation and precipitation processes in the natural waters are accompanied by co-precipitation and adsorption processes of different forms of phosphorus (Xiong and Peng 2011), a variety of adsorbents are added to water, for instance, iron oxide in order to increase their rate and to decrease the desorption of phosphorus compounds from the bottom sediments (Geelhoed et al. 1997; Zeng and Li 2004; Chitrakar et al. 2006). Other widely promoted adsorbents are lanthanum-modified bentonite (Haghseresht et al. 2009), natural zeolite (Tian et al. 2009; Dionisiou et al. 2013; Meng et al. 2013), mesoporus silicate material (Zhang et al. 2011), composite adsorbents containing two (or more) different metal oxides (Long et al. 2011; Li et al. 2014), and poly (vinyl alcohol) hydrogel beads with aluminum (Hui et al. 2014). Tian et al. (2009) recommended adsorption as one of the most effective removal processes for the low concentration of phosphorus.

Modern technologies for removal of phosphorus should be based on such materials which do not pose a toxicological threat to the environment, can be recovered and reused, as well as facilitate separation of the deposited phosphorus and its reuse as, e.g., fertilizer (de-Basahan and Bashan 2004; Tian et al. 2009). One of the possible solutions could be application of biosorbents or adsorbents obtained from biomass, which are more and more frequently in use for removing impurities from aquatic environments. Generally, biosorbents are cation exchangers binding in their structures metals, whereas adsorption of assimilable forms of biogenic elements requires their chemical modification. Scientific research has proven that building iron in structures of biosorbents improves qualities of sorptive phosphate in water solutions significantly (Unnithan et al. 2002; Eberhardt et al. 2006; Eberhardt and Min 2008). A considerable rise in adsorption of phosphate was observed after iron (III) ions (Fe (III))-treated; refined aspen wood fiber (Eberhardt et al. 2006; Eberhardt and Min 2008), coir path (Krishnan and Haridas 2008), eggshell waste (Mezenner and Bensmaili 2009), and *Staphylococcus xylosus* biomass (Aryal and Liakopoulou-Kyriakides 2011).

Substances of a similar ability to bind iron cations include alginate salts, which, when mixed with additives such as polyurethane (Sone et al. 2009), phosphorylated chitin (Jayakumar et al. 2009), or ferromagnetic citrate, form adsorbents of high adsorption capacity to metals such as nickel (Ngomsik et al. 2009; Jayakumar et al. 2009), zinc, copper (Jayakumar et al. 2009), and lead (Sone et al. 2009). Alginate acid is a natural biopolymer (anionic polysaccharide) extracted from algae. Alginate is nontoxic, non-immunogenic, and biodegradable and when connected with multivalent metals, e.g., calcium, forms structures of large specific areas (Bartkowiak and Hunkeler 1999; Hill and Khan 2008). The sorption properties of natural polysaccharide in the form of hydrogel capsules, for instance, calcium alginate have been confirmed, among others in the research concerning the purification of water from radionuclides (Mimura et al. 2001) and heavy metals (Nayak and Lahiri 2006; Hui et al. 2015).

Perfect adsorptive qualities of alginate in the form of small hydrogel capsules have been confirmed through researches concerning purifying water from heavy metals with radioactive characteristics (Mimura et al. 2001). One may assume that adsorbents obtained on the basis of alginate, having immobilized in it salts of multivalent metals, which are used in conventional methods of water treatment, might finally feature a strong affinity to phosphate. The purpose of this paper was to examine kinetics of bonds of phosphate at participation of the alginate/Fe (III) hydrogel capsules. Within the framework of conducted tests, it is planned to determine adsorption isotherms and check the impact of pH and temperature on this process.

## Materials and Methods

### Preparation of Alginate/Fe (III) Hydrogel Capsules

To receive the hydrogel capsules, an injection method with creation of a hydrogel polyelectrolyte complex—gelation of droplets of aquatic solution of polysaccharide with gelating salt. The polysaccharide, which was used for that purpose, was sodium alginate (Keltone HV, ISP-Germany), while the role of the gelating salt was played by iron (III) chloride (FeCl_3_ 6H_2_O) (EUROCHEM BGD, Tarnów, Poland) in the form of 0.155 M solution. The sodium alginate solution (1.5%) was obtained by mixing a sufficient quantity of sodium alginate with distilled water. In order to receive a solution containing fully hydrated polymer chains, the mixing process was carried out with use of a magnetic agitator for 24 h.

Hydrogel capsules of alginate/Fe (III) were received by dosing the sodium alginate in quantity of 5 mL each time with the classic 10-mL syringe (Polfa S.A., Lublin, Poland) ended up with a needle dia. 0.8 mm × 40 mm (TERUMO, Belgium) to 100 mL FeCl_3_ 0.155 M solution, at 2–3-min intervals. Time of the reaction after injection of the polysaccharide solution was 70 min. During forming the capsules, the FeCl_3_ solution was stirred continuously at 600 rpm. The capsules formed this way had a diameter of 2.5–3.5 mm.

### Analytical Methods

Phosphate concentration was measured by molibdenium blue colorimetric method (EN 1189, 1996), using a two-beam spectrophotometer Techcomp UV/VIS 8500, at wavelength 890 nm. Content of total iron (Fe) in the solutions was determined with the nuclear adsorption spectrometry, using a spectrometer ThermoElemental, Solaar S.

### Adsorption Kinetic Measurements

Kinetics of adsorption of phosphate was tested in deionized water (WD) and in two natural waters collected from eutrophic inland polymictic reservoirs (W1, W2), of which some selected qualitative indicators are shown in Table 1. Phosphate solutions (WD_P, W1_P, W2_P) with concentration 10 mg PO_4_ L^−1^ were prepared by dissolving KH_2_PO_4_ in the waters concerned. Then, 25 mL of prepared solutions and 20 capsules with alginate/Fe (III) were put into 50-mL Erlenmeyer flasks. Each portion of the capsules contained 2 mg of Fe. For each water, eight measurement series, repeated three times, were prepared. Changes in phosphate concentration, pH, and a general content of iron in aquatic phase were examined in three series of 1, 4, 7, 10, 24, 48, 96, 168 h. At the same time, content of phosphate was checked in referential tests on solutions without the capsules. The experiment was conducted in three temperatures at 4, 10, and 20 °C.

**Table 1:** Selected indicators of quality of the tested water

Water	pH	N_NH_4_ (mg L^−1^)	N_NO_2_ (mg L^−1^)	N_NO_3_ (mg L^−1^)	P_PO_4_ (mg L^−1^)
W1	7.61	0.58	0.004	0.17	0.02
W2	7.93	0.72	0.001	0.21	0.09

### Phosphate Adsorption Experiments

Phosphates adsorption characteristics were studied in a static batch system proposed by Naira et al. (1984). Using the deionized water (WD_P) and the two natural waters W1_P, W2_P, solutions of KH_2_PO_4_ with different content of phosphate (1, 2, 4, 10, 20, 40, 80 mg PO_4_ L^−1^) were prepared. Similarly to the previous experiment, some alginate/Fe (III) capsules were added. Adsorption isotherms were determined at 20 °C, changes in phosphate concentration were checked up after 48 h. The mixtures were shaken on a laboratory shaker for 2 h—in the beginning and in the end of the process of binding the phosphate.

Freundlich equation was fitted to phosphate adsorption by nonlinear regression:

$$ a\kern0.5em =\kern0.5em k\cdot {C}^n, $$

where *a* is the real adsorption, *C* is phosphate concentration in the equilibrium solution, *n* is the parameter that can be considered as a measure of adsorption intensity, and *k* is the parameter corresponding to the amount of phosphate adsorbed when *C* is equal to 1.

To evaluate the goodness of fit of the equation, the standard errors of the estimate were used along with *F* test.

### Effect of pH

In the deionized water, seven solutions of KH_2_PO_4_ were made at concentration 10 mg PO_4_ L^−1^, with various pH values 4, 5, 6, 7, 8, 9, 10, by adding appropriate quantities of HCl or NaOH in order to receive the required pH. The alginate/Fe (III) capsules were put to the solutions and after 48 h, quantity of PO_4_^3−^ ions was measured. The mixtures were shaken on a laboratory shaker for 2 h in the beginning and in the end of the process of binding the phosphate.

## Results and Discussion

### Phosphate Adsorption Kinetics

**Fig. 1:**
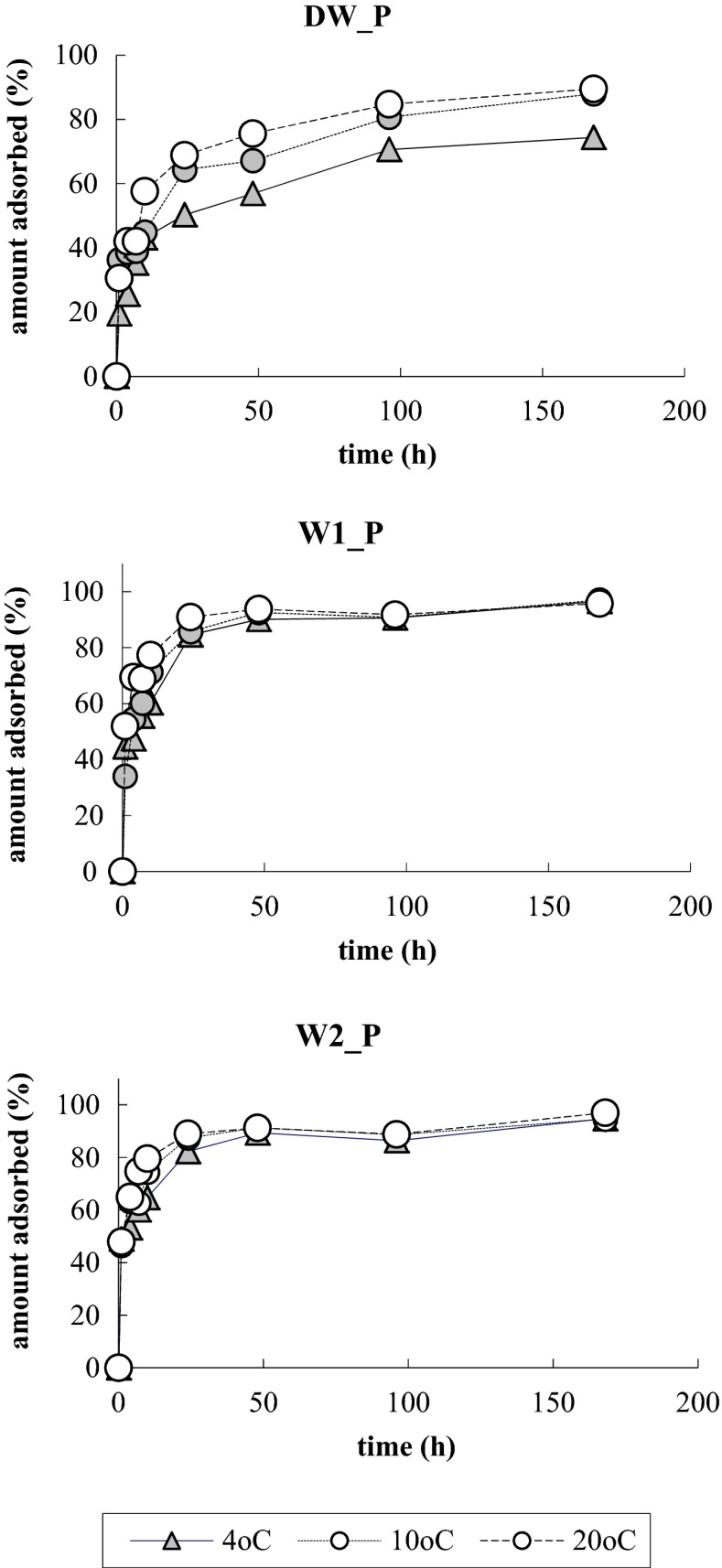
Changes in content of phosphate in distilled water DW_P and in natural waters W1_P and W2_P, at three temperatures

The results of tests on kinetics of phosphate inactivation at participation of the alginate/Fe (III) hydrogel capsules, at the temperatures of 4, 10, and 20 °C showed that quantity of adsorbed phosphate grew with time (Fig. 1). Most of the phosphate was adsorbed from their solution in distilled water (DW_P) after 10 h, and from the solutions in natural waters within no more than 4 h. After 24 h, from the DW_P solution, 61% of phosphate was removed, and from the natural water solutions W1_P and W2_P 87, it was 86%, respectively. After 48 h, practically in all the waters, an equilibrium was reached, and only insignificant changes were observed. After 7 days of the experiment, at 20 °C, 89.5% of phosphate was removed from DW_P, and 95.8 and 97% were removed from the natural water solutions W1_P and W2_P. In the distilled water, the pace of phosphate removal increased along the growth of temperature, similarly as in the case of adsorption of phosphate in natural sediments (Zhou et al. 2005) and on iron oxide (Zeng and Li 2004). In the natural waters, the temperature affected the process of phosphate removal at participation of the alginate/Fe (III) capsules from the solutions in the initial phase only (within the first 24 h); after stabilization, no differences in quantities of adsorbed phosphates were recorded.

The presented results show that the rate of inactivation of phosphate at participation of the alginate/Fe (III) capsules is higher and more efficient, when the adsorption comes from a solution of phosphate in natural waters than from their solution in distilled water. This can be related to the presence of ions existing in natural waters, e.g., calcium ion, which can affect the sorptive qualities of the capsules. Additionally, there can be a process of releasing Fe (III) to the solution due to ion exchange with ions of sodium or potassium, which are characterized by a higher affinity for carboxyl groups than, e.g., Fe (III). That is frequently the case in arrangements used for bio-immobilization, where there are complexes of alginate with calcium cations and wherein an environment with concentration of sodium cations similar to so called physiological salt, which is abt. 155 mM, a gradual exchange of calcium cations to sodium cations takes place.

**Fig. 2:**
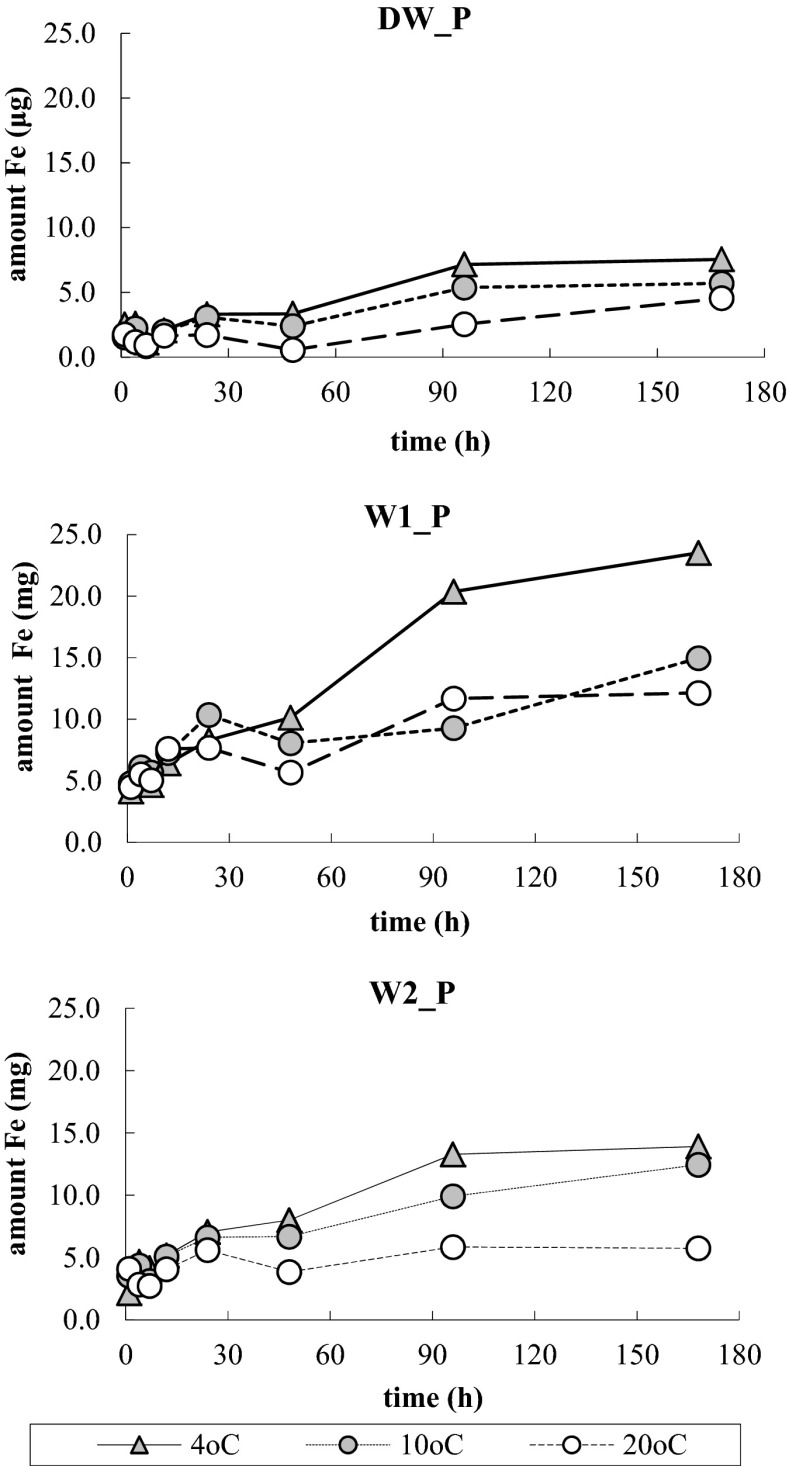
Changes in content of general iron in aquatic solutions of phosphate in 25 mL of distilled water DW_P and in 25 mL of natural waters W1_P and W2_P, at three temperatures

After introduction of the capsules to all the used types of water, within 1 h only, the following quantities of Fe (III) were released: 1.93 μg for solution DW_P; 4.48 μg for W1_P and 3.26 μg for W2_P (Fig. 2). During the next 4 h, content of iron in water DW_P dropped below 1 μg in a test, whereas content of iron in the natural waters increased. On the next stage of the process, almost in all cases, an increase of iron in the aquatic phase and stabilization of the equilibrium between the content of iron in aquatic phase and the hydrogel phase were observed. In all instances, a substantial influence of the temperature on the rate of Fe (III) release to the solution was seen—the rate decreased as the temperature increased. After completion of the experiment, the most of Fe (III) was release to solution W1_P, less to W2_P, and the least to WD_P; at 4 °C, these values were respectively 23.50, 13.91, and 3.71 μg.

**Fig. 3:**
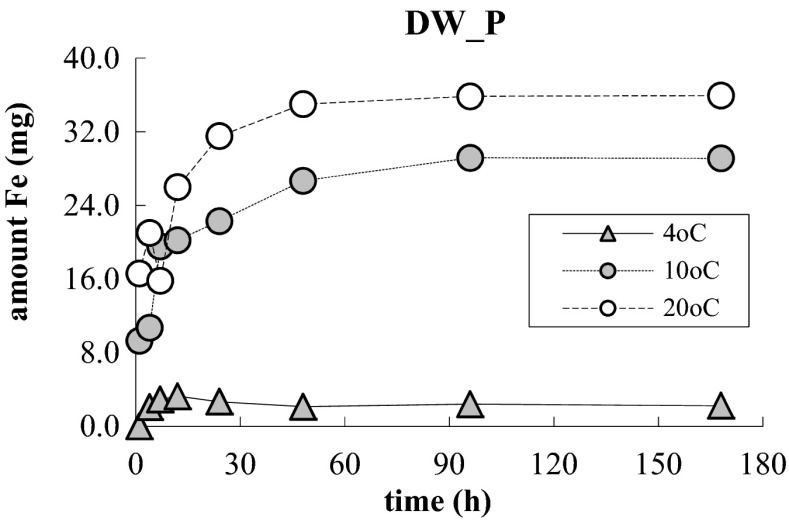
Changes in content of general iron in 25 mL of distilled water, at three temperatures

**Fig. 4:**
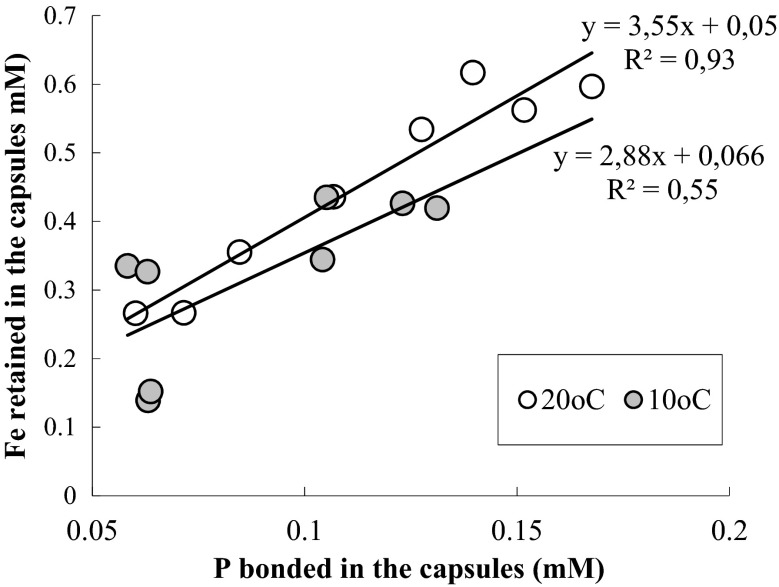
Dependence between the content of iron, which was retained inside the capsules at the presence of PO_4_^3−^ dissolved in the distilled water and the quantity of P bonded in the capsules at subsequent temporal measurements, at the two temperatures

In the same conditions, rate of releasing of Fe (III) to distilled water without participation of phosphate (DW) was measured, and the results are shown in Fig. 3. The process rate increased along with the temperature growth; at 10 and 20 °C, it was several times higher than at 4 °C. In this test, quantity of the released Fe (III) grew for 10 and 20 °C with time much faster than it happens at the presence of phosphate. As early as after 24 h of the process, at all the temperatures, stabilization occurred, and after 7 days, quantity of Fe was in the samples as follows: at 4 °C—2.2 μg, at 10 °C—29.1 μg, and at 20 °C—35.9 μg. This shows that phosphate get bonded with Fe (III) in the capsules, thus making it impossible to release those cations to the aquatic phase. This is confirmed by some strong linear correlations (Fig. 4), calculated for the dependence between the quantity of Fe (III), which was retained inside the capsules at presence of phosphate dissolved in distilled water and the quantity of phosphate bonded inside the capsules in subsequent temporal measurements, at 10 and 20 °C.

**Fig. 5:**
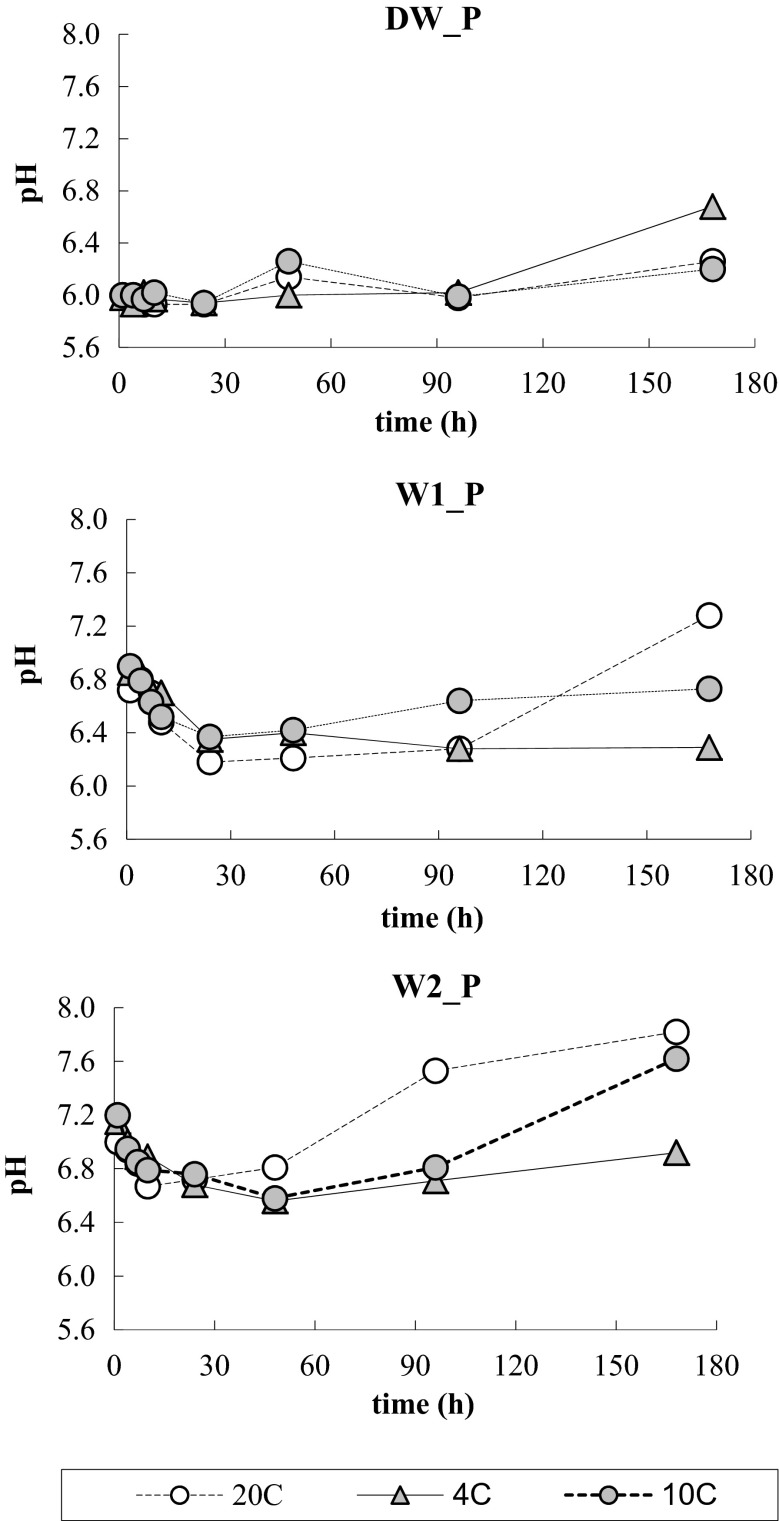
Changes in pH of the tested solutions during the phosphate removal process, at three temperatures

Value of pH in the solution of phosphate in water DW_P before application of the capsules was 6.00 and within the initial 24 h did not practically change, to increase after 7 days to 6.68 at 4 °C and to 6.20 and 6.26, respectively, at 10 and 20 °C (Fig. 5). Stronger changes in pH during the process were observed in solutions W1_P and W2_P, where, before application of the capsule, pH was respectively 6.67 and 7.00. Within the first 24 h, at 20 °C, a drop of pH value to 6.18 in W1_P and to 6.67 in W2_P was recorded, followed by a growth to 7.28 and 7.82, respectively, which values are approximate to the values the waters had before application of the capsules (Table 1). At the other temperatures, amplitudes of changes were lower and the smallest changes were seen for 4 °C.

The process of removal of phosphate at participation of alginate/Fe (III) capsules may combine two processes: adsorption on a large specific area of alginate salts, strengthened by the presence of Fe (III) in its structure, and a process of precipitation of phosphate in the aquatic phase with Fe (III), which get released from the capsules to the solution. Adsorption of phosphate in the capsules is indicated by significant differences between the rate and quantity of Fe released from the capsules to water WD_P (Figs. 2 and 3). Much larger quantities of Fe released to the DW water, compared to quantities of this metal released to solution WD_P, give evidence that phosphate combine with Fe (III) adsorbed in alginate, due to which their quantity released to the solution decreases. The process of precipitation of phosphate is signaled by a faster and more efficient process of removing these ions from the natural water solutions comparing to the solution of these ions in the distilled water (Fig. 1). As a result of ion exchange in the natural waters, a more intensive desorption of iron from the capsules to the liquid phase may take place than in the case of the distilled water (Fig. 2). With dissolved phosphate, the ions of Fe (III), which are present in water, may form sparingly soluble salts. This is indicated by the changes in pH, which were observed during the process (Fig. 4). The reduction of pH within the first 24 h in the natural waters might be attributed to reactions of hydrolysis of Fe (III), which deliver hydrogen ions. This is confirmed by the fact that in that period, no pH changes were observed in the phosphate solution in the distilled water, where content of iron and rate of the phosphate removal process were much lower.

The different influence of the temperature on the rate of iron desorption from the capsules to distilled water WD (Fig. 3) and to solution of phosphate (Fig. 2), as well as the important positive linear correlations (Fig. 4), calculated for the dependence between quantity of Fe (III), which was retained inside the capsules at the presence of phosphate dissolved in distilled water, and quantity of phosphate bonded inside the capsules shows that the processes taking place inside the capsules play a more important role than those in the aquatic phase. Quantity of Fe (III) released to the distilled water only increased along with the growth of temperature, while efficiency and rate of the process of releasing Fe (III) to phosphate solutions decreased with the growth of temperature. Having assumed that the whole iron contained in the aquatic phase is combined with phosphorus in the form of iron (III) phosphate, based on the quantity of iron determined in the aquatic phase and the quantity of phosphates removed after 7 days of the process running at 20 °C, as well as on a stoichiometric calculation of precipitation reactions, simple estimations of participation of individual mechanisms in the entire processes were made. During removal of phosphate from WD_P, about 1.3 % of these ions got precipitated and the remaining part was adsorbed in the capsules, whereas in the natural waters participation of precipitation in the whole phosphorus inactivation process reached 19.4 % in water W1_P and 19.8 % in water W2_P.

### Phosphate Adsorption Isotherm

**Fig. 6:**
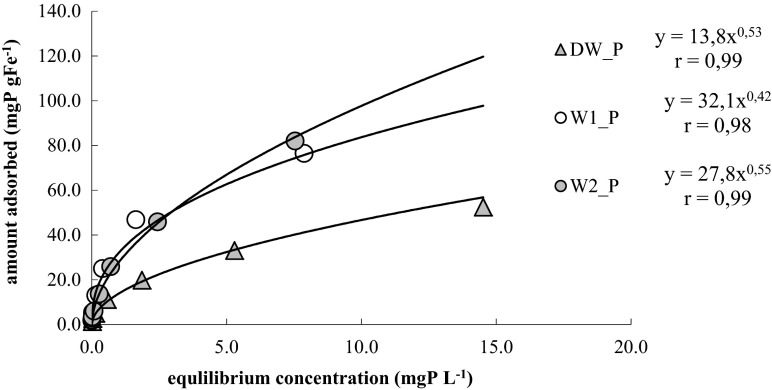
Isotherm of phosphate ion adsorption on the alginate/FeCl_3_ capsules in distilled water DW_P and in natural water W1_P and W2_P, at temperature 20 °C

According to Giles classification (Giles et al. 1974), the received form of the isotherm indicates that it is an isotherm class L (Fig. 6), characteristic for the arrangements free from competitive interactions between the adsorbent and the solvent. The same form of isotherm was received by authors examining the process of phosphorus adsorption on various adsorbents, e.g., goethite (Chitrakar et al. 2006; Nowack and Stone 2006), wood fiber treated with carboxymethyl (Eberhardt et al. 2006; Eberhardt and Min 2008), and surfactant-modified natural zeolite (Dionisiou et al. 2013). In the course of tests on phosphate adsorption on a mixture of iron oxide and gypsum, an adsorption isotherm class S was obtained, as characteristic for arrangements where the solvent is strongly adsorbed (Bastin et al. 1999)

Parameter *n* of the isotherms were quite similar to one another; the highest value was calculated for W2_P (*n* = 0.55). Parameter *k* reflecting the process of adsorption from natural waters W1_P and W2_P amounted to 32.8 and 27.8, respectively, and in both cases was over two times higher than the constant *k* calculated for the adsorption process conducted from distilled water, for which *k* = 13.8. Differences among these parameters might be related to the presence of other ions in the natural waters, which affect the process of releasing Fe (III) to the solution, and, at the same time, the phosphate ion inactivation process. For other sorbents, adsorptive characteristics of phosphate from natural waters are weaker than in the distilled water, e.g., in case of adsorption in geothite or akaganeite (Chitrakar et al. 2006). The received very high values of parameter *k* should not be compared directly to the parameter *k* calculated in works describing adsorption of phosphorus with other adsorbents containing iron, as their authors calculate the quantity of adsorbed phosphorus per 1 g or per 1 m^2^ of specific area of the entire adsorbent. In this paper, because of the lack of opportunity to weigh the adsorbent mass, the quantity of phosphorus removed from the solution is expressed by conversion to 1 g of iron contained in the alginate/Fe (III) hydrogel capsules. Therefore, the received values of parameter *k* are sometimes dozens of times higher than those found in the literature on this type of processes. Nevertheless, they indicate that by using much smaller quantities of iron, being the sole component of the adsorbent concerned, which can pose a toxic threat to the environment, it is possible to remove similar or even higher quantities of phosphorus than it happens in the case of other adsorbents containing iron. Parameter *n*, calculated for the process of adsorption of phosphates from the solution in distilled water, was 0.53 (Fig. 6) and is higher than the parameters received for adsorption with other adsorbents containing iron, e.g., mixture of iron oxides, geothite, akaganeite, lignocellulose saturated with iron, and wood fiber treated with carboxymethyl, where the values remained within the range between 0.11 and 0.42 (Zeng and Li 2004; Chitrakar et al. 2006; Nowack and Stone 2006; Eberhardt et al. 2006; Eberhardt and Min 2008).

### Effect of pH on Phosphate Adsorption

**Fig. 7:**
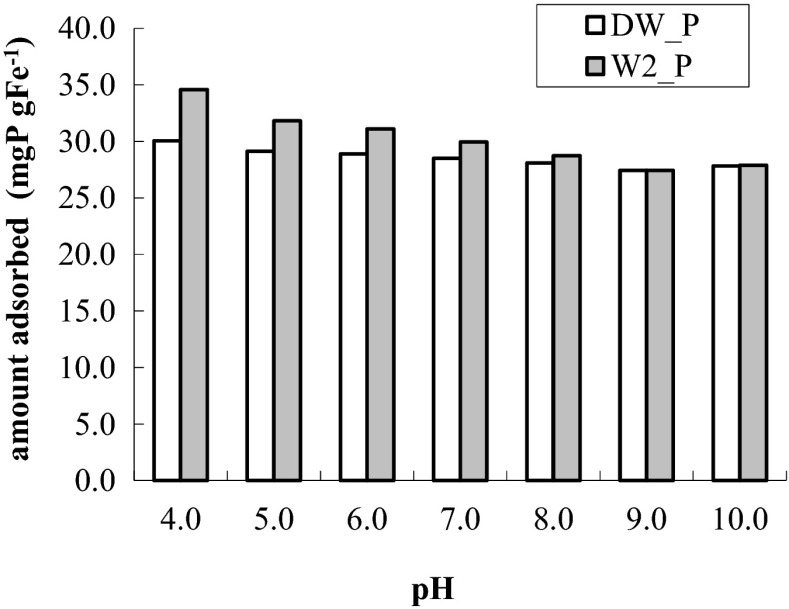
Influence of pH in the solution on the process of removing phosphate from their solution in distilled water DW_P and in natural water W2_P

Impact of pH of the solution on the process of releasing phosphates from their solution in distilled water DW_P and from the solution in natural water W2_P is shown in Fig. 7. Along with the growth of pH, quantity of removed phosphates decreased. Reduction of adsorption capacities with growth of pH in a solution of P was observed in the phosphate adsorption process on other adsorbents, e.g., aluminum hydroxide (Tanada et al. 2003), iron (III) oxide (Zeng and Li 2004), and goethite (Geelhoed et al. 1997); the presented results show that adsorbent alginate/FeCl3 removes phosphate at a wide range of pH: from 4 to 10.

## Conclusions

Rate of phosphate inactivation at participation of the alginate/FeCl_3_ capsules is higher and more efficient, when the adsorption proceeds from solutions of phosphate in natural waters rather than in distilled water. In distilled water, the rate of removing phosphorus from the solution grew with the temperature growth. In natural waters, temperature affected the course of removing phosphorus from solutions at participation of the alginate/FeCl_3_ only during the first 24 h, and after reaching a balance state, no differences in quantities of adsorbed phosphates were recorded. The course of adsorption of phosphates and desorption of iron to the aquatic phase, as well as the changes in pH, indicate that the process of removing PO_4_^3−^ at participation of the alginate/FeCl_3_ capsules consists generally in adsorption, on a large specific area, alginate salts strengthened by Fe (III) present in their structure, whereas simultaneously, but to a much smaller extent, a process of precipitation of phosphate with Fe (III) released from the capsules to the solution may take its place. Parameters calculated with Freundlich isotherm equation, which was used to describe removal of phosphates from aquatic solutions at participation of the alginate/FeCl_3_, show that by using much less Fe (III) it is possible to the same or bigger quantities of phosphorus than it is the case with other adsorbents containing iron. Adsorbent alginate/FeCl3 removes phosphate within a wide range of pH: from 4 to 10. The conducted tests show that the alginate/FeCl_3_ capsules possess a strong ability to inactivate phosphate in aquatic solutions. The proposed adsorbent can be a subject of farther application studies in the view of using it for cleaning and reclamation of water.
